# A Miniature Pockels Cell with Novel Electrode Geometry

**DOI:** 10.3390/s90705298

**Published:** 2009-07-06

**Authors:** Slobodan J. Petricevic, Pedja Mihailovic, Jovan Radunovic

**Affiliations:** Faculty of Electrical Engineering / Bulevar Kralja Aleksandra 73, 11000 Beograd, Serbia; E-Mails: pedjamih@gmail.com (P.M.); radunovic@etf.rs (J.R.)

**Keywords:** Pockels cell, electrode geometry, delta-sigma, Bi_12_GeO_20_, fiber optic sensor

## Abstract

This paper discusses important elements of the Pockels sensing cell design. A novel electrode geometry is analyzed in order to obtain maximum sensitivity response from Pockels crystals (Bi_12_GeO_20_). This neither transversal nor truly longitudinal geometry, results in electrical field distribution along the sensing beam path that provides high modulation depth. Demonstrated performance level is in agreement with theoretical studies. Delta-sigma polarization detection method allows high linearity of the detector transfer function and measurement independent on laser intensity variations. Channel gain equalization process necessary for accurate delta-sigma normalization is provided by a walk off prism.

## 1. Introduction

Increasing need for electric power demands larger capacity of electric power systems. Hence, transmission voltage increases as well as the sizes of voltage and current transformers. Fiber-optic sensors have the potential to replace voltage and current transformers by offering several advantages: immunity to electromagnetic interference, high dynamic range, compact design, impossibility of explosion and wide bandwidth [1–4]. On the other hand, in order to replace existing technology, fiber-optic sensors manufacturers must have lower costs and improve temperature compensation techniques.

The principle of the fiber-optic current sensor (FOCS) is to measure the magnetic field caused by the sensed current using Faraday effect. The fiber-optic voltage sensor (FOVS) is based on the Pockels effect and measures electric field to calculate the voltage. Used together they constitute an electric power meter for supreme diagnostics of electric power systems [5]. It is a commercial benefit and a technological simplification to use the same optical crystal for both current and voltage sensing.

Our crystal of choice is bismuth germanium oxide, Bi_12_GeO_20_ (BGO). BGO is transparent over a wide wavelength range and is a low-cost material readily available without optical defects. BGO crystals have cubic symmetry, space group 23 and are without birefringence but they possess optical activity. BGO shows Faraday rotation with a Verdet constant of approximately 70 rad/Tm. Absence of birefringence is crucial for successful FOCS construction [6]. Since BGO exhibits diamagnetic properties Faraday rotation temperature compensation of FOCS can be realized [7].

This paper presents construction and calibration of a low cost Pockels cell with a BGO crystal, small in dimensions and ready to be incorporated in polarimetric extrinsic fiber-optic voltage sensors. The electrooptical coefficient of BGO is *r_41_*[10^−12^ m/V ] = 3.67, *λ* = 633 nm [8] and that is sufficient for FOVS construction. Additionally, normalized temperature dependence of the BGO optical activity is negative [9] and can be used to compensate for the temperature dependence of the Pockels effect in the crystal [10].

Ohtsuka and Shimomura have optimized length and configuration of electrooptic crystals to increase sensitivity [11]. However, further improvement can be made by electrode geometry that provides optimal electrical field distribution along the light beam path enabling increased modulation depth. This paper proposes a novel, neither transversal nor truly longitudinal, electrode geometry that provides better signal to noise ratio for higher accuracy measurements.

## 2. Pockels Sensing Setup

**Figure 1.**
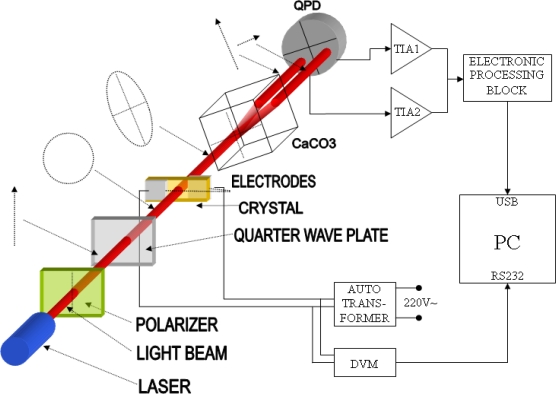
Sensing setup.

For the light source we chose a 1 mW, 632.8 nm wavelength He-Ne laser. A polarizing prism (PP) was placed in front of the laser. This decreases the light beam irradiation but suppresses noise, since polarization fluctuations are converted to light irradiation fluctuations. A zero order quarter-wave plate follows the polarizing prism with the fast axis at a 45° angle with respect to the polarizer transmission axes. Circularly polarized light impinges on the Pockels cell and becomes elliptically polarized due to the electric field. The birefringent crystal (CaCO_3_) with fast and slow axes parallel to the major and minor axes of the refraction ellipse is placed behind the Pockels cell. It spatially divides the laser beam into two components with mutually orthogonal polarizations. Irradiations of both beams depend on the light source intensity in the same way. Using this fact we can calculate the retardance Φ that is not dependent on the intensity of the light source by difference-over-sum method. This concept is illustrated in Figure 1. Beam irradiations are measured using two segments of a quadrant photodiode (4QPD), followed by a dual transimpedance (DUAL TIA) stages. Stages are identical, based on dual operational amplifier, having the same transimpedance gains.

In order for this sensing system to achieve the desired accuracy, it is required that the detection gains of two beams be identical. Equalization is accomplished by adjusting the parameters of the transimpedance stages with both crystals removed from the beam path. Loss imbalance between the two channels after separation of two orthogonally polarized light beams is minimized by using a highly birefringent CaCO_3_ crystal instead of a polarizing prism and making the separate optical paths parallel. This fact also enables the use of the quadrant photodiode thus matching optoelectronic conversion gains as much as possible.

## 3. Pockels' Cell Construction and Modeling

**Figure 2.**
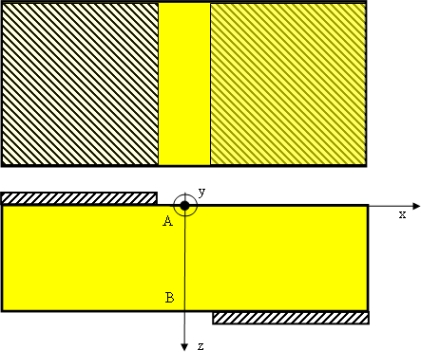
Geometry of electrodes.

The task of the Pockels cell design is to provide maximum possible retardance for the applied voltage. Retardance depends on the choice of electro-optic crystal and on the geometry of the Pockels cell electrodes. For reasons stated in the introduction, we have selected a bismuth germanium oxide – Bi_12_GeO_20_ – BGO crystal as the electro-optic crystal. It has cubic symmetry, space group 23 and it is without birefringence, but possesses optical activity. The BGO crystal is cut parallel to the (1 1 0) plane and is 2 mm thick. We assume that the z axis is orthogonal to this plane and that the light wave travels along the z axis. In order to optimize geometry of the Pockels cell electrodes let us now consider a Pockels cell with neither transverse nor truly longitudinal configuration as displayed in Figure 2.

In such electrode geometry the electrical field contains *E_x_* and *E_z_* components. Since the point group of BGO is 23 electrooptic tensor has the form:

(1) $$\underline{r}=\left[\begin{array}{ccc} 0 & 0 & 0\\ 0 & 0 & 0\\ 0 & 0 & 0\\ r_{41} & 0 & 0\\ 0 & r_{41} & 0\\ 0 & 0 & r_{41} \end{array}\right],$$

and index ellipsoid is:

(2) $$\frac{x^{2}+y^{2}+z^{2}}{n_{o}^{2}}+{2r}_{41}E_{x}\mathrm{yz}+2r_{41}E_{z}\mathrm{xy}=1$$

To find refractive indices in xy plane we set z = 0.

(3) $$\frac{x^{2}+y^{2}}{n_{o}^{2}}+{2r}_{41}E_{z}\mathrm{xy}=1$$

Rotation of x and y axes around the z axis by 45° gives principal indices of refraction *n_x'_* and *n_y'_*:

(4) $$\begin{array}{l} x=x^{\prime }\text{cos}\frac{\pi }{4}+y^{\prime }\text{sin}\frac{\pi }{4}\\ y=-x^{\prime }\text{sin}\frac{\pi }{4}+y^{\prime }\text{cos}\frac{\pi }{4} \end{array}$$

(5) $$\left(\frac{1}{n_{o}^{2}}-r_{41}E_{z}\right)x^{\prime }+\left(\frac{1}{n_{o}^{2}}+r_{41}E_{z}\right)y^{\prime }=1$$

(6) $$\left(\frac{1}{n_{o}^{2}}-r_{41}E_{z}\right)=\frac{1}{{n^{\prime }}_{x}^{2}}\Rightarrow {n^{\prime }}_{x}=\frac{n_{o}}{\sqrt{1-r_{41}E_{z}n_{o}^{2}}}\Rightarrow {n^{\prime }}_{x}\approx n_{o}\left(1+\frac{1}{2}n_{o}^{2}r_{41}E_{z}\right)$$

(7) $$\left(\frac{1}{n_{o}^{2}}+r_{41}E_{z}\right)=\frac{1}{{n^{\prime }}_{y}^{2}}\Rightarrow {n^{\prime }}_{y}=\frac{n_{o}}{\sqrt{1+r_{41}E_{z}n_{o}^{2}}}\Rightarrow {n^{\prime }}_{y}\approx n_{o}\left(1-\frac{1}{2}n_{o}^{2}r_{41}E_{z}\right)$$

(8) $$d\Phi =\frac{2\pi }{\lambda_{0}}\left({n^{\prime }}_{x}-{n^{\prime }}_{y}\right)\mathrm{dz}=\frac{2\pi }{\lambda_{0}}n_{o}^{3}r_{41}E_{z}\left(z\right)\mathrm{dz}$$

And the total retardance is:

(9) $$\Phi =\frac{2\pi }{\lambda_{0}}n_{o}^{3}r_{41}\int_{A}^{B}E_{z}\;\left(z\right)\mathrm{dz}=\frac{2\pi }{\lambda_{0}}n_{o}^{3}r_{41}\int_{A}^{B}\vec{E}\left(z\right)d\vec{l}=\frac{2\pi }{\lambda_{0}}n_{o}^{3}r_{41}U_{\mathrm{AB}}.$$

Since the BGO crystal is a linear medium, there is a linear relationship between voltages *U_AB_* and measured voltage *U: U_AB_* = *MU*, 0 < *M < 1*.

By introducing this linear relation in (9) one obtains:

(10) $$\Phi =\frac{2\pi }{\lambda_{0}}n_{o}^{3}r_{41}\mathrm{MU}=\pi \frac{U}{U_{\pi }}M$$

The BGO crystal possesses following important electro optical properties at *λ_0_* = 633 nm [8]:

1. *r_41_* is 3.67·10^−12^ m/V
2. *n_0_* = 2.55

There is up to 5% difference in these quantities between various crystal samples.

Thus the half-wave voltage *U_π_* is:

(11) $$\frac{\lambda_{0}}{{2n}_{o}^{3}r_{41}}=U_{\pi }=5200V$$

and it depends on the crystal type but is invariant on the crystal size and shape of electrodes.

Factor M is dependent on the electric field distribution and can be optimized by shaping the geometry of the electrodes in such a fashion to maximize the scalar product under the integral in equation 9. For each different crystal lengths there is an optimal geometry of electrodes and electrodes efficiency can be compared using factor M so we'll call it efficiency factor.

Since optical activity prevents an increase of the cell sensitivity by increasing the crystal size [11] we have chosen a BGO crystal that is 2 mm thick.

The lower limit of the cell cross section size is set by the light beam used in this experiment. For the Uniphase model 1101P He-Ne laser used, beam waist diameter is *w_0_* = 0.63 mm and beam diameter *w* at the sensing point *z* = 0.5 m is:

$$w=w_{0}\sqrt{1+{\left(\frac{z}{z_{0}}\right)}^{2}},\;z_{0}={\frac{\pi nw_{0}^{2}}{\lambda }}_{,w=0.9\mathrm{mm}}$$

For these dimensions parallel-plate capacitor approximation is not valid and instead of the standard longitudinal modulation geometry we examined a geometry presented in Figure 2. We have made an experimental performance comparison between a conventional longitudinal modulation Pockels cell and this novel asymmetric configuration.

## 4. Signal Processing

**Figure 3.**
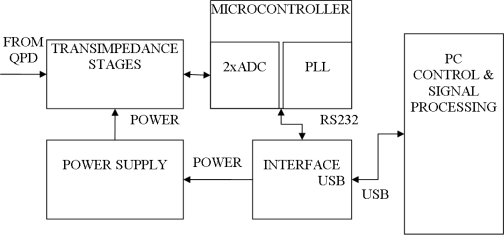
Block diagram of the signal processing circuit.

Signal processing is accomplished along the lines of the block diagram shown in Figure 3. Following discussions relates to this diagram. Information on the measured high voltage is present in the two low power photocurrents coming from two quadrants of the zero biased four quadrant photodiode (QPD). These photocurrents are amplified and converted to voltage by symmetrical transimpedance stages. Operational amplifiers used in these stages are mounted on the same PCB at the optical setup end. Resulting voltages are fed to two analog to digital converters (2xADC) inside a SiLabs C8051F064 microcontroller (MCU). Converters operate at 10 kSPS with 16 bits of resolution and store acquired waveforms in an onboard memory. Memory is organized as triple buffers. Data transfers take place simultaneously with data acquisition process via USB interface. This enables data acquisition of newer signal periods while older periods are transferred to the PC for processing. The microcontroller is connected to a RS232 to USB converter (INTERFACE USB) that provides a USB link to the PC in the form of the virtual COM port. USB power supply (5 V DC) is converted to ± 10 V DC used to power operational amplifiers in transimpedance stages and other analog components. This function is accomplished by the POWER SUPPLY block.

Of particular importance is the phase locked loop (PLL) block used to lock the ADC sample rate to power line frequency. This is necessary since frequency locking of the sampling rate eliminates leakage and picket fencing in the frequency domain. PLL is constructed using a voltage controlled oscillator (VCO) as a frequency generator and multiplication ratio is determined by counting the number of high frequency pulses in one signal period (20 ms). The VCO input is adjusted using a DA converter to establish the required multiplication. Once this ratio is established, the MCU checks the mentioned count and determines its rate of change, in order to anticipate next accurate values for VCO input. This process is continuous, and is used to hold the PLL in the locked state.

Gains of transimpedance stages are programmable, coarse gain setting is accomplished using digital potentiometers for transimpedances and fine gain control is accomplished using ADC calibration. Gain controls are necessary in order to equalize gains (slopes of the light to voltage transfer functions) of the two independent sensing channels in delta-sigma normalization method.

**Figure 4.**
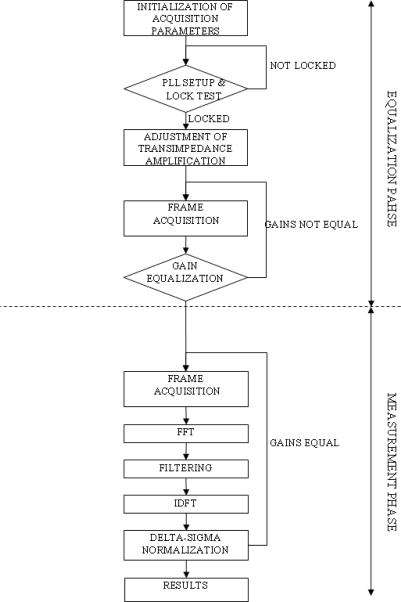
Signal processing algorithm.

The PC controls execution of all steps in the algorithm by commands sent via a USB link to the onboard MCU. MCU executes commands and responds with data streams containing waveforms of sampled values. The signal processing algorithm is presented in Figure 4. Initial control steps establish acquisition rate, number of samples per period, number of periods per frame and starting the PLL block. The PLL then runs until it locks, and further steps are introduced to check the PLL stability and jitter. Once the necessary conditions for accurate sensing are established, the PLL is left under the control as described to maintain it in the locked state. Then, the light source is switched off and offsets in transimpedance stages are eliminated by applying compensation voltages to passive inputs of operational amplifiers. Transimpedances of TIAs are then set to accomplish equal DC response (coarse gain control) of both channels. The ADC fine gain control is used to equalize DC responses below level of detection (angular error of 1 mdeg). This ends the calibration procedure and channels are now considered to be both equalized and calibrated to the necessary degree of accuracy.

There follows a continuous loop consisting of frame acquisition, processing, filtering and result calculation. The PC instructs the MCU to acquire a new frame, check for completion flag, and retrieves the frame to the internal PC memory. The frame is then processed to deal with ADC output formats and discrete Fourier transform (DFT) is then calculated. Operations take place in baseband, one frequency bin is 50 Hz wide, there being a total of 200 bins per waveform for a 10 kHz sample rate. DFT obtained in this way is filtered by removing all frequency components apart from the base frequency and is reverse processed using inverse discrete Fourier transform (IDFT) to obtain filtered time domain waveforms. These waveforms are then displayed for observation purposes.

Results are calculated using the magnitudes of both channels base frequency DFT bins and a rms value is obtained from them. These rms values are used as inputs in delta sigma equation and the resulting angle is displayed on the screen.

## 5. Experimental Results

With channels gains equalized, axes of the birefringent crystal aligned for the maximum response and in the case when retardance is much smaller than optical activity, output signal voltages *U_1/2_* of the transimpedance stages are:

(12) $$U_{1/2}=\frac{k\Gamma_{0}}{2}\left(1\pm \Phi \frac{\text{sin}\;\rho }{\rho }\right)$$

where *Γ_0_* is the beam irradiation, *ρ* is the optical activity and *k* is a constant that models optical losses and optoelectronic conversion efficiency [11–13].

The difference over sum response is:

(13) $$R_{\Delta /\Sigma }=\frac{U_{1}-U_{2}}{U_{1}+U_{2}}=\Phi \frac{\text{sin}\;\rho }{\rho }=\pi \frac{M}{U_{\pi }}\frac{\text{sin}\;\rho }{\rho }U=\mathrm{cU}$$

**Figure 5.**
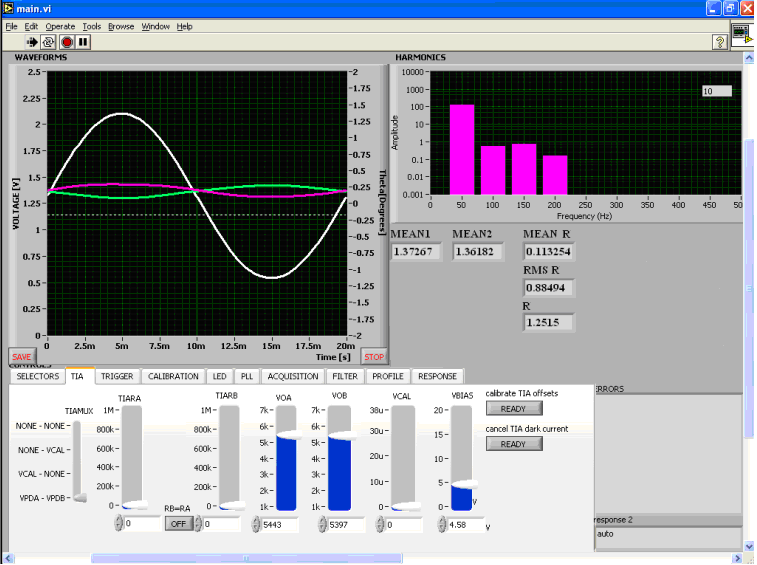
Measurement screen for excitation voltage of 71.5 V.

Calibration of the Pockels cell was performed experimentally, by measuring the response *R_Δ/Σ_* obtained from Δ/Σ sensing method, as described above. The magnitude of the AC excitation on the crystal electrodes was varied by means of the autotransformer and measured with the voltmeter connected to a PC using a serial port. The form of the measurement screen can be seen in Figure 5 together with results obtained for an excitation voltage of 71.5 V (amplitude). The green and magenta signals represent output voltages of the transimpedance stages (left axis). The white signal represents the response *R* (right axis, denoted Theta on the screen). All signals are functions of time denoted by the Time axis in [s]. Harmonics diagram displays frequency content of the response signal (digital filter implements a cut off above 200 Hz). Numeric displays present mean response value MEAN R (0.113253 deg), response (RMS R) calculated from the frequency bin at 50 Hz, and response (R) value calculated from the waveform, also in degrees. Lower part of the screen contains functions for controlling the electronic signal processing circuit necessary to implement gain equalization.

**Figure 6.**
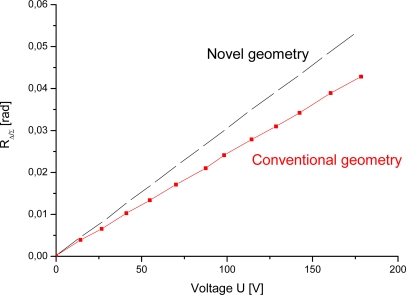
Calibration graph.

Our experiments show that coefficient *M* for 2 mm thick crystal is higher for the geometry shown in Figure 2. For both configurations the efficiency factor *M* can be obtained from Figure 6 using a linear fit of the experimental data. For the case of asymmetric electrodes, *c* is 3.0325 × 10^−4^ [V^−1^]. Since optical rotatory power at *λ* = 632.8 nm is 18°/mm, then optical activity *ρ* = 36°. Therefore efficiency factor *M* is 0.537. For the case of conventional longitudinal electrode configuration, *c* is 2.39258 × 10^−4^ [V^−1^], therefore the *M* is 0.423.

## 6. Conclusion

A new Pockels cell electrode geometry has been investigated in order to increase the modulation depth and decrease cell dimensions. The mathematical model presented shows that modulation depth depends not only on electrooptical properties of the crystal, but also on the electrodes position and shape. This dependence is more significant at lower crystal thickness to beam diameter ratio. The same mathematical model is applicable for comparison of various electrode configurations using the efficiency factor M. A novel geometry Pockels cell, 2 mm thick and with 6 x 4 mm cross section, was constructed and experimentally compared with a standard longitudinal Pockels cell of the same dimensions. Procedure for two channels gain equalization, crucial for laser intensity and polarization fluctuation suppression, in Δ/Σ normalization method is described in detail. Accurate signal processing in frequency domain is accomplished by dedicated PLL circuit that establishes fixed relationship between sampling and signal frequency. This novel geometry of electrodes increases the slope of the transfer function by 1.27-fold.
